# A three generation study of the mental health relationships between grandparents, parents and children

**DOI:** 10.1186/1471-244X-13-299

**Published:** 2013-11-09

**Authors:** Kirsten J Hancock, Francis Mitrou, Megan Shipley, David Lawrence, Stephen R Zubrick

**Affiliations:** 1 Telethon Institute for Child Health Research, The University of Western Australia, PO Box 855, West Perth, WA 6872, Australia

**Keywords:** Intergenerational transfer, Mental health, Children and families

## Abstract

**Background:**

It is well known that children of parents with mental illness are at greater risk of mental illness themselves. However the patterns of familial mental health problems across multiple generations in families are less clear. This study aimed to examine mental health relationships across three generations of Australian families.

**Methods:**

Mental health data, along with a range of family demographic information, were collected from over 4600 families in *Growing Up in Australia: The Longitudinal Study of Australian Children*, a nationally representative cohort study. The social and emotional wellbeing of two cohorts of children aged 4–5 years and 8–9 years was measured using the parent-rated Strengths and Difficulties Questionnaire (SDQ). The mental health of mothers and fathers was measured using the Kessler 6-item K6 scale, and the mental health history of maternal and paternal grandmothers and grandfathers was measured using a dichotomous parent-report item. Multivariate linear regression analyses were used assess the relationships between grandparent and parent mental health and child social and emotional wellbeing at ages 4–5 years and 8–9 years.

**Results:**

Both cohorts of children had greater mental health distress with higher SDQ scores on average if their mother or father had a mental health problem. For children aged 8–9 years, a history of mental health problems in maternal grandmothers and grandfathers was associated with higher SDQ scores in grandchildren, after controlling for maternal and paternal mental health and other family characteristics. For children aged 4–5 years, only a mental health history in paternal grandfathers was associated with higher SDQ scores.

**Conclusions:**

The mental health histories of both parents and grandparents play an important role in the social and emotional wellbeing of young children.

## Background

Children of parents with mental illness are at significantly greater risk for multiple psychosocial problems [1-4]. These children are also more likely to experience developmental delays, lower academic competence and difficulty with social relationships [5-7]. Most inter-generational research has focused on documenting parent–child mental health associations, as parents are typically the most proximal and influential people in a child’s development, particularly during the early years. By virtue of these parent–child mental health relationships, it follows that the mental health histories of grandparents could have some influence on the mental health of their grandchildren. This may occur through a direct relationship with the child, or through their indirect relationship with the child via the parents. Many mental disorders are considered to have a hereditary component to them [8-10], but families also share environments and experiences in addition to their genes.

Studies examining multi-generational mental health relationships have received greater attention in recent years [11-17]. Of the scant literature examining these relationships, there is wide variation in methodologies and in diagnostic tools used to assess mental health problems, including the completeness of family pedigree, the use of direct and indirect assessments and differing interview or questionnaire methods. For example, some of the studies collected mental health data for either maternal or paternal family members [12,14] where others collected data for both [11,13]. These variations contribute to a generally mixed and inconsistent range of findings, which we briefly review below.

In the most recent study, Cents et al. [11] found grandparent lifetime anxiety and depression predicted both internalizing and externalizing problems in their 3 year old grandchildren. These associations were independent of parent psychopathology, but were found only when using mother’s, but not father’s, report of children’s internalizing and externalizing problems. Cents et al. also found that for each additional grandparent with a lifetime history of psychiatric disorder, the risk of both internalizing and externalizing problems for children increased by a factor of 1.6–1.7, independent of parental psychiatric disorder.

Using a variety of mental health measures included in the 1970 British Cohort Study, Johnston et al. [17] found a grandparent-parent and parent–child intergenerational mental health correlation of about 0.2. The correlation between grandparent and grandchild mental health was weaker, and the effect persisted across different frequencies of grandparent-grandchild contact. There was no significant grandparent-grandchild correlation once parent mental health was taken into account.

Pettit et al. [14] examined the relationships between grandparent major depressive disorder (MDD), parent psychiatric history, and internalizing problems in children aged 2 to 18 years. They found no evidence of a direct relationship between grandparent MDD and grandchild internalizing problems, however children had higher levels of internalizing problems when both a parent and a grandparent had experienced depression.

Olino et al. [13] examined the extent to which MDD in parents and grandparents, assessed using an array of clinical diagnostic instruments, was related to children’s internalizing and externalizing problems. Parent MDD, grandparent MDD and the interaction of both were associated with children’s internalizing problems at age two. In contrast to Pettit et al. [14], Olino et al. found the presence of MDD in both the parent and grandparent generation did *not* convey additional risk over and above the direct effect of parent and grandparent MDD on children’s internalizing problems. The presence of grandparent MDD was only associated with internalizing problems in the absence of MDD in both parents.

Weissman et al. [16] used a clinical sample of grandparents to examine the impact of grandparent and parent MDD upon assessments of children’s psychopathology. They found both parent and grandparent MDD was related to children’s psychopathology in middle childhood. They also found children were at greater risk of a mood disorder if they had both a parent and grandparent with MDD. This finding mirrored the results of an earlier study by Warner et al. [15].

Finally, Hammen, Shih and Brennan [12] collected data from a community sample of adolescents, mothers and maternal grandmothers, to examine mental health transmission over three generations, finding that both maternal grandmother and maternal depression had an influence on adolescent mental health. They were unable to assess the influence of paternal depression on children’s mental health outcomes.

These multi-generation studies used different sampling methods and diverse measures of mental health, often collected at a variety of developmental epochs. Several used small samples [15,16], had limited measures of family socio-demographic environments [12,13], or lacked mental health data for the full family pedigree [14,17]. Only one study had complete information on socio-demographic environment and the full family pedigree [11]. Despite these differences, the studies collectively indicate that the mental health histories of grandparents appear to have an effect on children’s mental health, and particularly so in families where mental health problems exist for multiple family members in multiple generations. However, because the pattern of results differs across the studies, it is difficult to ascertain how this occurs. Direct and indirect influences may operate through shared environments, genes, or an interaction of the two.

Grandparents may directly influence the mental wellbeing of their grandchild, which is partially supported by the studies demonstrating a grandparent-grandchild mental health association even in the absence of mental health problems in parents [11,13,15,16]. An example of a direct pathway might be in the way that a grandparent with anxiety disorder interacts with their grandchild. When spending time together, the child may learn through the grandparent’s disposition and behavior that the world should be viewed a frightening place. The child may then learn to become anxious and frightened in general. In such cases, we would expect that the grandparent-grandchild mental health relationship would be stronger in families where children are in frequent contact with their grandparents. Only one of the previous studies examined this idea [17], with results showing that the strength of the grandmother-grandchild mental health association was the same across families with differing frequencies of contact with grandmothers, however the grandmother-grandchild relationship was no longer significant once parent mental health was included in the model.

Such a result suggests that the influence of mental health problems in grandparents could operate indirectly, where the grandparent’s mental health influences parents’ mental health which, in turn, influences the child’s mental health. For example, a grandparent with an anxiety disorder might influence the way their own child views the world. When that child grows up and becomes a parent, at greater risk of anxiety disorder themselves, the parent in turn influences the emotional wellbeing of the grandchild. Thus, the role of grandparents operates via the parent generation. This pathway would be supported by results where the grandparent-grandchild association is no longer observed once parent mental health is accounted for, though this pattern has only been shown in one of the multi-generational studies [17]. Finally, both direct and indirect mechanisms are likely to be in play. Where this occurs we might expect to see an attenuated, though not entirely diminished, relationship between grandparent and grandchild mental health once the mental health of parents has been accounted for. We might also observe an interaction between parent and grandparent mental health on child wellbeing, where the risk of mental health problems in children would increase in terms of both generational proximity and intergenerational burden, which has also been supported by the studies reviewed earlier [11,14-16].

This study examined multi-generational mental health associations in Australian families in order to further understand the nature of intergenerational mental health associations. We used data from *Growing Up in Australia, the Longitudinal Study of Australian Children* (LSAC) to investigate how mental health problems in two generations of family members impacted upon children’s subsequent social and emotional wellbeing. Data were collected for over 4,600 children, and included mental health data for mothers, fathers and maternal and paternal grandparents of these children. The LSAC also collects substantial contextual information, including the amount of face-to-face contact children have with their grandparents, along with the family’s social and demographic characteristics. Such data have been relatively rare in other multi-generational studies.

We hypothesized parents would be at greater risk of mental health problems if their own parents (hereafter to be called grandparents) had a history of mental health problems. Second, children would be more likely to have poorer social and emotional wellbeing if they had a grandparent with a history of mental health problems, however this grandparent-child association would be attenuated relative to the more proximal parent–child estimate of association. Finally, we hypothesized that children would have poorer social and emotional wellbeing if they had multiple family members with mental health problems, relative to children with only a parent or grandparent with a mental health problem.

## Methods

### Study design and population

This study used data from LSAC, a nationally representative study of Australian children and their families. LSAC data were initially collected in 2004 from two cohorts of children; 5,107 infants aged 3–19 months (B-cohort) and 4,983 children aged 4 years 3 months–5 years 7 months (K-cohort). The same children were revisited in 2006 (Wave 2) and 2008 (Wave 3) when the B-cohort children were aged 2–3 years and 4–5 years, and the K-cohort children were aged 6–7 years and 8–9 years. Of the 10,090 families that participated at Wave 1, 9,070 (89.9%) participated at Wave 2 and 8,718 (86.4%) participated at Wave 3. Data from both cohorts were used for this study. Wave 3 data were primarily used for this study, and supplemented with grandparent mental health data, which were collected at Wave 2.

The LSAC employed a two-stage clustered sample design, with Australian postcode area as the primary sampling unit. The sampling frame was extracted from the Medicare Australia enrollment database [18]. Approximately one in ten Australian postcode areas were randomly selected and children then were randomly selected within postcode areas ensuring only one child per household was selected (the study child). At the time of sample selection, more than 90% of infants born in Australia were enrolled on the Medicare database by 4 months of age, and 98% by 12 months [19]. The initial response rate at Wave 1 was 54.8% (n = 5,107) for the B-cohort and 47% (n = 4,983) for the K-cohort. The initial sample was broadly representative of the Australian population of families with children in the LSAC age groups compared with 2001 Census data, but slightly under-representative of families who were single-parent, non-English speaking, living in rental properties or living in remote areas [20].

### Data collection methods

In-home interviews were held at each wave with the primary carer residing with the study child (Parent 1). Additionally, Parent 1 and the second carer residing with the study child (Parent 2, where there was a second parent) were provided with self-complete questionnaires at each wave. Approximately 75% of self-complete surveys were returned by Parent 1 and 2 at Waves 2 and 3. In most cases Parent 1 was the biological mother (97%), and Parent 2 was the biological father (92-96%). Information about the biological heritage of the study child’s grandparents was not collected. Further details on response rates for interviews and survey instruments are available elsewhere [21]. Formal consent to take part in the study was obtained from Parent 1. Approval for the study was granted by the Australian Institute of Family Studies Ethics Committee.

### Mental health measures

#### Grandparents

The mental health histories of maternal and paternal grandparents of the study child were collected retrospectively from the Parent 1 and Parent 2 self-complete questionnaires at Wave 2. Parents were asked to recall “did your father (or father figure) suffer from nervous or emotional trouble or depression?” followed by the same question about their mother (or mother figure). Parents could respond “yes” or “no”.

#### Parents

The Kessler K6 scale [22] assessed mothers’ and fathers’ level of non-specific psychological distress in the self-complete questionnaire. Parents were asked how often in the past 4 weeks they had felt nervous; hopeless; restless or fidgety; that everything was an effort; so sad that nothing would cheer you up; or worthless, and responded on a 5-point scale from 0 = all of the time to 4 = none of the time. Responses were reverse coded, summed and adjusted to generate a total score ranging from 0 to 24, where higher scores represented greater levels of non-specific psychological distress. Scores of 13 or above indicate a probable serious mental illness [23]. As the LSAC is a non-clinical sample very few parents fell above this cut-point. In concert with other studies [24,25] we used a lower cut-point of 8+ to classify parents as having elevated non-specific psychological distress. Data from the National Survey of Mental Health and Wellbeing in Australia has shown that 53% of Australian adults with a score of 8–12 on the K6 had an International Classification of Disease-10 (ICD-10) anxiety or depressive disorder as diagnosed by the Composite International Diagnostic Interview [26]. Although the K6 scale refers to the 30 days prior to the survey, 89% of adult respondents who had an ICD-10 diagnosis of anxiety or depressive disorder and scored between 8–12 on the K6 had their first onset of symptoms more than 5 years prior to the survey. The K6 scale and using a cut-point of 8 may therefore be considered a reliable and valid indicator of non-specific psychological distress in Australian adults.

#### Children

The Strengths and Difficulties Questionnaire (SDQ) [27] was used to measure emotional and behavioral problems when the B cohort were 4–5 years and the K cohort 8–9 years. The SDQ is a 25-item scale comprising 5 sub-scales covering peer relationships, conduct problems, hyperactivity, emotional problems and pro-social behavior. The SDQ was collected via the Parent 1 self-complete questionnaire. Total scores were calculated by summing scores on the peer, conduct, hyperactivity and emotional problems sub-scales. Scores could range from 0 to 40, with higher scores representing poorer functioning.

### Other measures

A unique feature of the LSAC methodology was the inclusion of a measure of child contact with their grandparents. Parent 1 reported how often the study child would “get together with, see, or spend time with your parents” and their partner/spouse’s parents, with responses including: don’t have; no contact; rarely; a few times a year; at least every month; at least every week; and, every day.

Other covariates known to be associated with social and emotional wellbeing problems in children included: *Family type* comprised two categories; children in two-parent blended families and two-parent original families. *Socioeconomic Position* (SEP) was derived from equivalized household income, parent’s level of education and occupational prestige [28]. Families in the lowest 25% of SEP were categorized as disadvantaged. *Mother’s age* at the birth of her first child was included as: less than 20 years; 20–24 years; and 25 years or more. Finally, ongoing medical conditions in either parent and medical conditions in the child that had lasted 6 months or more were also included.

In total, mental health data were collected on 7,366 children, 7,447 mothers, 5,421 fathers, 6,723 maternal grandmothers, 6,696 maternal grandfathers, 5,930 paternal grandmothers and 5,931 paternal grandfathers. There were 4,606 cases with complete mental health information for all family members. The characteristics of families with complete data were different to those with incomplete data. Cases with incomplete mental health data were over-represented in terms of social and emotional wellbeing problems in the study child (17.1% vs. 12.7%), single-parents (29.6% vs. 2.4%) and blended families (7.2% vs. 3.3%), socio-economic disadvantage (40.4% vs. 19.6%) and younger first-time mothers (15.7% vs. 5.1%).

### Statistical analysis

All analyses were weighted using survey weights and adjusted to account for the complex survey design. Prevalence rates of elevated psychological distress in parents according to the mental health status of grandparents were assessed using survey weighted cross-tabulation and Rao-Scott adjusted Chi-Square analyses. Mean differences in children’s SDQ scores according to the mental health status of parents and grandparents were initially assessed using weighted univariate regression analyses. Multivariate linear regression analyses were used to examine the relative effects of parent and grandparent mental health status on mean SDQ scores, after controlling for the family and demographic covariates specified above. SAS 9.3 was used to conduct all analyses.

## Results

Descriptive statistics and sample characteristics are provided in Table 1 for each of the cohorts. With the exception of contact with grandparents, there were no substantial differences between the cohorts on any of the characteristics included in the study. Contact with both maternal and paternal grandparents occurred more frequently for the children aged 4–5 years than those aged 8–9 years.

**Table 1:** Sample characteristics for the B cohort (4–5 years) and K cohort (8–9 years)

	**B cohort**	**K cohort**
	**N = 2676–4253**	**N = 2636–4196**
**Variable**	**% (N)**	**% (N)**
**Child SDQ mean (SD)**	8.5 (0.1)	7.8 (0.1)
**Parent mental health**		
Mother has elevated psychological distress	10.6 (350)	14.0 (458)
Father has elevated psychological distress	9.5 (235)	11.5 (279)
**Grandparent mental health**		
Maternal grandmother, with MH history	24.8 (818)	25.1 (801)
Maternal grandfather, with no MH history	16.5 (540)	15.1 (479)
Paternal grandmother, with no MH history	20.3 (608)	18.4 (524)
Paternal grandfather, with no MH history	11.7 (348)	11.9 (342)
**Child contact with maternal grandparents**		
Don’t have	2.5 (96)	5.1 (192)
No contact	2.9 (110)	4.2 (157)
Rarely	9.0 (344)	11.8 (444)
A few times a year	18.9 (719)	19.4 (729)
At least every month	18.7 (712)	21.7 (817)
At least every week	38.8 (1479)	30.5 (1148)
Every day	9.2 (352)	7.4 (280)
**Child contact with paternal grandparents**		
Don’t have	6.3 (238)	10.1 (375)
No contact	5.1 (195)	6.4 (238)
Rarely	13.5 (514)	14.7 (547)
A few times a year	21.8 (826)	22.4 (835)
At least every month	22.0 (835)	22.5 (838)
At least every week	26.8 (1017)	19.5 (729)
Every day	4.6 (173)	4.5 (168)
**Family type**		
Two-parent original	83.4 (3653)	76.6 (3318)
Two-parent blended	2.8 (107)	7.0 (252)
Lone-parent family	13.8 (455)	16.3 (581)
**Mothers age at birth of first child**		
Less than 20 years	11.0 (321)	10.4 (332)
20-24 years	22.2 (866)	23.9 (904)
25+ years	66.8 (3066)	65.7 (2960)
**Socioeconomic Position**		
Disadvantaged	30.6 (1031)	30.9 (1031)
Not disadvantaged	69.4 (3218)	69.1 (3162)
**Medical problem in child**		
No	89.8 (3850)	90.7 (3827)
Yes	10.2 (403)	9.3 (369)
**At least one parent has medical problem**		
No	86.7 (3731)	85.1 (3602)
Yes	13.3 (522)	14.9 (594)

MH = Mental Health.

### Grandparent–parent

A higher proportion of mothers had elevated psychological distress when maternal grandmothers had a history of mental health problems compared to those who did not (17.4% vs. 8.7%), and when maternal grandfathers had a history of mental health problems compared to those who did not (17.9% vs. 9.5%, unadjusted analyses, see Table 2). Similarly, a higher proportion of fathers had elevated psychological distress when paternal grandmothers had a history of mental health problems compared to those who did not (15.0% vs. 8.7%) and likewise for paternal grandfathers (16.1% vs. 9.1%).

**Table 2:** **Percentage of mothers and fathers (95**% **confidence interval) experiencing elevated psychological distress (K6 > =8), by grandparent mental health history (unadjusted analyses)**

**Mental health history in grandparent**		**N (%)**	**Parents with elevated psychological distress (%)**	**95% CI**	**Test statistic**	**p-value**
**Mothers**						
Maternal grandmother	No	4441 (74.8)	8.7	7.8–9.6	*χ*^2^ (1) = 87.1	<.001
	Yes	1498 (25.2)	17.4	15.2–19.5		
Maternal grandfather	No	4996 (84.4)	9.5	8.6–10.3	*χ*^2^ (1) = 58.3	<.001
	Yes	926 (15.6)	17.9	15.1–20.8		
**Fathers**						
Paternal grandmother	No	3661 (80.1)	8.7	7.7–9.7	*χ*^2^ (1) = 32.5	<.001
	Yes	908 (19.9)	15.0	12.6–17.5		
Maternal grandfather	No	4025 (88.1)	9.1	8.1–10.1	*χ*^2^ (1) = 25.3	<.001
	Yes	544 (11.9)	16.1	12.6–19.5		

B and K Cohort figures are not shown separately, as the figures for each cohort are highly similar.

### Parent–child

In unadjusted analyses, mean SDQ scores for both cohorts of children were higher when either the mother or father had elevated psychological distress (see Table 3). Across both cohorts, average SDQ scores were 11.3 and 7.7 respectively for children of mothers with and without elevated psychological distress (t_(926.9)_ = 15.3, p < .001). Likewise, mean SDQ scores were 9.7 and 7.5 for children of fathers with and without elevated psychological distress (t_(570.2)_ = 7.4, p < .001).

**Table 3:** Unadjusted mean SDQ scores for the B and K cohort children, by presence of elevated psychological distress in mothers and fathers

**Elevated psychological distress in mother or father**		**N (%)**	**Mean SDQ**	**95% CI**	**Test statistic**	**p-value**
**B cohort (4–5 Years)**						
Mothers	No	3212	8.1	8.0–8.3	t(398.4) = 10.0	<.001
	Yes	334	11.4	10.8–12.0		
Fathers	No	2349	7.9	7.7–8.1	t(254.3) = 4.8	<.001
	Yes	217	9.8	9.0–10.6		
**K cohort (8–9 Years)**						
Mothers	No	3039	7.2	7.0–7.4	t(531.0) = 12.0	<.001
	Yes	440	11.2	10.7–11.7		
Fathers	No	2269	7.0	6.8–7.2	t(316.0) = 5.9	<.001
	Yes	267	9.6	8.8–10.4		
**Total**						
Mothers	No	6251 (89.0)	7.7	7.5–7.8	t(926.9) = 15.3	<.001
	Yes	774 (11.0)	11.3	10.9–11.7		
Fathers	No	4618 (90.5)	7.5	7.3–7.6	t(570.2) = 7.4	<.001
	Yes	484 (9.5)	9.7	9.1–10.3		

*T*-test assumes variances are unequal.

### Grandparent–child

Unadjusted analyses also showed that mean SDQ scores were typically higher for children whose grandparents had a history of mental health problems than those children whose grandparents did not, and this pattern was observed for both grandmothers and grandfathers on the maternal and paternal sides of the family (see Table 4). The effects were somewhat larger in the older cohort, with difference scores ranging from 0.8 to 1.6 points for the 8–9 year olds, and from 0.3 to 0.8 points for the 4–5 year olds. The effects were also stronger for maternal grandparents relative to paternal grandparents.

**Table 4:** Unadjusted mean SDQ scores, by cohort and grandparent mental health history

**Grandparent mental health history**		**N**	**Mean SDQ**	**95% CI**	**Test statistic**	**p-value**
**B cohort (4–5 Years)**						
Maternal grandmother	No	2194	8.1	7.9–8.3	t(1207.8) = 4.1	<.001
	Yes	751	8.9	8.5–9.2		
Maternal grandfather	No	2464	8.2	8.0–8.4	t(653.7) = 3.1	.002
	Yes	475	9.0	8.6–9.4		
Paternal grandmother	No	2083	8.0	7.8–8.3	t(914.4) = 1.7	.083
	Yes	562	8.3	8.0–8.7		
Paternal grandfather	No	2332	8.0	7.8–8.2	t(401.5) = 1.9	.052
	Yes	316	8.7	8.2–9.3		
**K cohort (8–9 Years)**						
Maternal grandmother	No	2159	7.3	7.0–7.5	t(1205.8) = 3.6	<.001
	Yes	736	8.1	7.7–8.5		
Maternal grandfather	No	2447	7.2	7.0–7.4	t(565.4) = 4.4	<.001
	Yes	436	8.8	8.2–9.3		
Paternal grandmother	No	2066	6.9	6.7–7.1	t(703.0) = 3.1	.002
	Yes	486	7.7	7.3–8.2		
Paternal grandfather	No	2233	6.9	6.7–7.2	t(396.7) = 2.2	.027
	Yes	318	7.7	7.2–8.3		
**Total**						
Maternal grandmother	No	4353	7.7	7.5–7.9	t(2419.2) = 5.4	<.001
	Yes	1487	8.5	8.2–8.8		
Maternal grandfather	No	4911	7.7	7.5–7.9	t(1219.2) = 5.4	.002
	Yes	911	8.9	8.5–9.2		
Paternal grandmother	No	4149	7.5	7.3–7.6	t(1612.2) = 3.7	<.001
	Yes	1048	8.0	7.7–8.4		
Paternal grandfather	No	4565	7.5	7.3–7.7	t(798.7) = 2.9	.004
	Yes	634	8.2	7.7–8.7		

*T*-test assumes variances are unequal.

### Multivariate analyses of intergenerational effects

Multivariate linear regression models were used to estimate differences in child SDQ scores according to the parent and grandparent mental health variables, after adjusting for child gender, face-to-face contact with maternal and paternal grandparents, family type, socioeconomic position, mother’s age at first birth, presence of a medical condition in the child and presence of a medical condition in either parent (see Table 5). Models are presented separately for each cohort, and include an interaction term for maternal and paternal mental health. The values in Table 5 provide the estimated difference in SDQ scores according to the variable included. For example, for the 4–5 year old cohort, SDQ scores were 1.82 points higher on average for children whose mothers had elevated psychological distress compared with children whose mother did not have elevated psychological distress. Children would score an additional 2.41 points if both parents had elevated psychological distress. For the 8–9 year old cohort, SDQ scores were 2.02 points higher on average if the mother had elevated psychological distress, and 1.30 points higher if fathers had elevated psychological distress, though there was no interaction between maternal and paternal mental health.

**Table 5:** Results from multivariate linear regression models estimating child SDQ scores, by child cohort

	**B cohort (4–5 years, n = 2081)**				**K cohort (8–9 years, n = 1988)**				**Total (n = 4069)**			
**Predictor**	**B**	**SE**	**p-value**	**95% CI**	**B**	**SE**	**p-value**	**95% CI**	**B**	**SE**	**p-value**	**95% CI**
Intercept	8.89	0.58	<.001	7.75–10.03	11.00	0.76	<.001	9.51–12.49	9.83	0.50	<.001	8.84–10.83
**Parent mental health status**												
Mother has MH problem	1.82	0.43	<.001	0.99–2.66	2.02	0.37	<.001	1.29–2.76	1.91	0.29	<.001	1.34–2.48
Father has MH problem	0.56	0.40	.169	-0.23–1.35	1.30	0.48	.007	0.35–2.25	0.94	0.33	.005	0.29–1.58
Mother x Father MH problem	2.41	0.98	.014	0.49–4.34	0.51	0.92	.577	-1.30–2.33	1.23	0.67	.067	-0.09–2.54
**Grandparent mental health history**												
Maternal grandmother has MH history	0.36	0.24	.126	-0.10–0.83	0.45	0.23	.048	0.01–0.90	0.41	0.17	.016	0.08–0.74
Maternal grandfather has MH history	0.31	0.28	.263	-0.23–0.85	0.62	0.30	.037	0.04–1.21	0.49	0.20	.015	0.10–0.89
Paternal grandmother has MH history	-0.13	0.21	.526	-0.55–0.28	0.46	0.28	.098	-0.09–1.01	0.21	0.18	.237	-0.14–0.56
Paternal grandfather has MH history	0.64	0.33	.049	0.01–1.28	0.12	0.33	.717	-0.53–0.77	0.36	0.27	.112	-0.09–0.81
**Contact with maternal grandparents**												
Infrequent vs no contact or none	0.55	0.41	.179	-0.26–1.36	-0.42	0.49	.391	-1.39–0.54	0.09	0.34	.794	-0.58–0.76
Frequent vs no contact or none	0.20	0.42	.625	-0.62–1.03	0.00	0.50	.997	-0.97–0.98	0.18	0.34	.604	-0.49–0.84
**Contact with paternal grandparents**												
Infrequent vs no contact or none	0.64	0.32	.045	0.02–1.28	0.73	0.37	.048	0.01–1.45	0.71	0.25	.005	0.21–1.20
Frequent vs no contact or none	0.47	0.31	.128	-0.14–1.07	0.35	0.33	.289	-0.30–1.01	0.49	0.24	.039	0.24–1.20

MH = Mental Health. Infrequent contact includes “rarely” and “a few times a year”. Frequent contact includes “at least every month”, “at least every week” and “every day”. Model adjusts for family structure, socio-economic position, mother’s age at birth of first child, presence of medical condition lasting 6 months or more in child, at least one parent has an ongoing medical condition and child gender, bold type p < .05. Interactions tested but found to be non-significant: mother has MH problem x maternal grandmother/grandfather has MH history; father has MH problem x paternal grandmother/grandfather has MH history; maternal grandmother/grandfather has MH history x contact with maternal grandparents; paternal grandmother/grandfather has MH history x contact with paternal grandparents.

After controlling for parental mental health and other covariates, a history of mental health problems in maternal grandmothers and grandfathers was associated with higher SDQ scores, but only for the 8–9 year old children. A history of mental health problems in paternal grandfathers was associated with higher SDQ scores for the 4–5 year old children. Children with infrequent or frequent contact with paternal grandparents had higher SDQ scores on average than children with no contact with their grandparents or those without grandparents, but no association was found for contact with maternal grandparents. Finally, a number of interactions were tested but found to be non-significant. These included interactions between parent and grandparent mental health, and grandparent mental health and contact with grandparents (see Table 5 legend for further detail).

## Discussion

It has only been in the last 10–15 years that research has extended beyond the association between parent and child mental health relationships to consider the link between the mental health of grandparents and grandchildren. The few studies on this topic have generally demonstrated that children of grandparents with a mental illness such as MDD or anxiety disorder are at greater risk of social and emotional wellbeing problems [11-16]. However, there is considerable variability in the results of these studies, and the nature of the grandparent-grandchild mental health association remains unclear. Whilst some studies have found that poor mental health in grandparents predicts poor mental health in grandchildren, independently from parent mental health status [11,13,16], others did not find any such relationship [14,17].

Some of the multi-generational mental health literature has provided support for a cumulative or dose–response pattern, finding children are at greater risk of developing mental health problems if there is a history of mental health difficulties in both the parent and grandparent generations, compared to history in just the parent or grandparent generation [14-16]. However, this pattern has not always emerged [13] or been tested [11,12,17] in these multi-generational mental health studies.

There are two key features of this study that merit comment. First, though we found a significant association between a history of mental health problems in maternal grandparents and child SDQ scores, there was no evidence of a cumulative impact on children from having a parent and a grandparent with mental health difficulties, as noted by a the lack of significant interactions between parent and grandparent mental health. However, we did find a cumulative impact of having both a mother and a father with elevated psychological distress for the 8–9 year old children. That is, there appears to be some cumulative impact of having two parents with elevated psychological distress, but no cumulative relationship of children having worse outcomes if they had a grandparent and parent with a mental health problem.

This pattern has not been a consistent result in other multi-generational studies. Of the studies examining the cumulative impact of familial mental health problems, three found evidence of a cumulative relationship [14-16] related to psychopathology and internalizing problems in children. One study did not find a cumulative relationship [13] though this study examined behavior outcomes of children. Cents et al. [11] also found a cumulative impact in the grandparent generation, where the risk of both internalizing and externalizing problems in grandchildren increased for each successive grandparent with an anxiety or depressive disorder.

The second notable finding of this study is that a history of broadly defined nervous and emotional mental health problems in grandparents is associated with elevated SDQ scores, even after controlling for elevated psychological distress in parents. This result suggests that children are susceptible to the mental health influence of grandparents, and supports the notion of some direct impact of grandparental mental health on children. This result is consistent with the other multi-generational studies that have used either community or clinical samples along with comprehensive, validated diagnostic instruments [11-13,15,16].

We also found a different pattern of results for the 4–5 year olds compared to the 8–9 year old children. With the exception of paternal grandfathers, there were no significant differences in SDQ scores according to the history of mental health problems in grandparents for the 4–5 year old children. For children aged 8–9 years however, a history of mental health problems in either the maternal grandmother or grandfather was associated with significantly higher SDQ scores. It is difficult to determine why different patterns would be observed for each cohort. It is possible that the older children, by virtue of their age, have had more opportunity to interact with their grandparents, and the association may reflect the accumulation of exposure to potential mental health problems in grandparents, though we are not able to offer additional evidence in support of this. It is also possible that the SDQ measure is more sensitive to problem behavior amongst older children. That average SDQ scores were somewhat higher in the younger cohort of children, particularly amongst those whose parents did not have elevated psychological distress (Table 3) would support this explanation. As such, any relationship between grandparent mental health and child SDQ scores would become more evident when children are older. We do note that some of the other multi-generational studies that used different measures observed a grandparent-grandchild mental health relationship for children as young as two or three years of age [11,13].

The grandparent-grandchild relationship persisted after controlling for elevated psychological distress in parents, irrespective of the amount of contact with grandparents. This result could suggest that the relationship does not necessarily occur through the face-to-face relationship, but rather through other inherited pathways, be they genetic or environmental and family contexts. For example, mental health problems are more prevalent in disadvantaged families, and previous research has consistently shown there are also strong intergenerational associations in education, occupation, and family income [29,30]. For many families, the persistence of mental health problems across generations is likely to be tied to these inherited family contexts. However, in assessing the ability of the contact measures to shed light on how these pathways might operate, it is important to note the limitations of the items measuring contact with grandparents. For example, the question did not specify which grandparent the child spent time with. It is possible that grandchildren spend more time with one grandparent than another, particularly in circumstances where grandparents had separated or one had passed away. As such, the measure may not be a reliable indicator of the amount of time spent with the grandparent who had the history of mental health problems and these results should therefore be interpreted with some caution. There are also many reasons that children may have limited contact with their grandparents that could relate to their social and emotional wellbeing. Grandparents may be deceased, or live a long distance away, and for some parents a lack of family support may impact on their wellbeing. There may also be relationship difficulties, perhaps related to mental health, that mean parents feel it is in the best interest of the children that they should have limited contact with their grandparents. As such, it is difficult to hypothesize exactly what role contact with grandparents would have on the grandparent-grandchild mental health association.

There are limitations to this study. The mental health measures used, while commonly employed in large scale epidemiological studies, are not direct measures of psychiatric morbidity. The broad-based developmental focus of the LSAC precluded using diagnostic tools for study children and parents. The questions posed to parents about grandparent’s mental health were very broad and represent secondary and retrospective recall rather than a direct measure on the grandparent. The grandparent mental health questions directed to parents were in the context of their family life when they were growing up. Therefore, for grandparent mental health, the timing, severity and persistence of any nervous or emotional problems is unclear. We cannot determine whether these problems occurred before or after the study child was born and therefore what sort of influence the grandparent may have had on the study child. In this respect, finding grandparent-parent and grandparent-grandchild associations at all demonstrates how strong and pervasive these relationships can be. Also, the relationships documented in this study are observational and as such we cannot make any causal conclusions, particularly in relation to parental mental health. For example, the presence of mental health problems in both parents and children may reflect aspects of their shared environment that have not been accounted for, such as a death in the family or income instability.

For the majority of families, the mother was the informant for her child, her own and the maternal grandparent mental health measures, and the father was the informant for his own and paternal grandparent mental health measures. Therefore, we cannot discount that there may be an informant bias. Such a bias may inflate these intergenerational mental health relationships to be stronger than they might otherwise be, and could be an explanation as to why stronger grandparent-grandchild mental health relationships were found for maternal grandparents than paternal grandparents. However, given some of our findings, it is unlikely that the results we observed were solely due to informant bias. For example, we show that elevated psychological distress in fathers and paternal grandparents was associated with child wellbeing even though mothers completed the SDQ for the majority of children. Additionally, though we used Wave 3 measures for child wellbeing and parent mental health, questions relating to grandparent mental health were collected at Wave 2. Finally, another research study using the LSAC data has shown similar patterns for the intergenerational impacts of joblessness and separation on children [31]. In that study, joblessness and separation in grandparents were associated with lower social and emotional wellbeing, literacy and numeracy achievement in grandchildren. These significant family events are examples of reports that are less biased or subjective than the reports of mental health history used in this study. However, similar patterns were observed, and as such, we have reasonable confidence that results we observed are not solely the result of informant bias.

In acknowledging these limitations, there are several strengths to this study that should also be noted. In addition to the availability of data on both parent and grandparent mental health, our study offers further advances with respect to the strength of the LSAC data. The large sample size and the wide range of socio-demographic characteristics included in this study has alleviated concerns other studies have faced around poor statistical power and allowed us to control for potential socio-demographic confounders, which previous studies could not achieve. Though contact with grandparents did not modify the grandparent-grandchild association, assessing the amount of face-to-face contact children had with their grandparents is particularly important as the ability of grandparents to directly influence the mental wellbeing of their grandchildren would be constrained in situations where grandparents see their grandchildren less frequently. These relationships require further research with more informative measures as to the nature of the relationship that grandparents have with their grandchildren.

## Conclusions

With the noted limitations in mind, we can conclude from this study that assessments of children’s social and emotional wellbeing should take into account a full family history of mental health problems. Grandparent mental health relates to parental mental wellbeing, which in turn is associated with the wellbeing of children. However, there is also a direct mental health relationship between grandparents and grandchildren independent of mental health difficulties in parents.

## Abbreviations

LSAC: Longitudinal study of Australian children; MDD: Major depressive disorder; SDQ: Strengths and difficulties questionnaire; SEP: Socioeconomic position.

## Competing interests

The authors declare that they have no competing interests.

## Authors’ contributions

KH, FM and MS designed the study. KH conducted the analysis and drafted the initial manuscript. FM, DL, MS and SZ provided feedback on initial analysis. All authors contributed to editing and revising the manuscript. All authors read and approved the final manuscript.

## Pre-publication history

The pre-publication history for this paper can be accessed here:

http://www.biomedcentral.com/1471-244X/13/299/prepub
